# Targeted Therapies in Early Stage NSCLC: Hype or Hope?

**DOI:** 10.3390/ijms21176329

**Published:** 2020-08-31

**Authors:** Alex Friedlaender, Alfredo Addeo, Alessandro Russo, Vanesa Gregorc, Diego Cortinovis, Christian D. Rolfo

**Affiliations:** 1 Oncology Department, University Hospital of Geneva, 1205 Genève, Switzerland; alex.friedlaender@hcuge.ch (A.F.); alfredo.addeo@hcuge.ch (A.A.); 2 Medical Oncology Unit, A.O. Papardo, 98158 Messina, Italy; ale.russo1986@gmail.com; 3 Department of Oncology, Division of Experimental Medicine, IRCCS San Raffaele, 20132 Milan, Italy; vanesa.gregorc@hsr.it; 4 SC Medical Oncology, Azienda Socio Sanitaria Territoriale (ASST) H S Gerardo Monza, 20900 Monza, Italy; d.cortinovis@asst-monza.it; 5 Thoracic Oncology Department and Early Phase Clinical Trials Section, School of Medicine, University of Maryland, Baltimore, MD 20742, USA

**Keywords:** NSCLC, targeted therapy, early stage, EGFR, ALK, osimertinib

## Abstract

Non-small-cell lung cancer (NSCLC) represents roughly 85% of lung cancers, with an incidence that increases yearly across the world. The introduction in clinical practice of several new and more effective molecules has led to a consistent improvement in survival and quality of life in locally advanced and metastatic NSCLC. In particular, oncogenic drivers have indeed transformed the therapeutic algorithm for NSCLC. Nearly 25% of patients are diagnosed in an early stage when NSCLC is still amenable to radical surgery. In spite of this, five-year survival rates for fully resected early stage remains rather disappointing. Adjuvant chemotherapy has shown a modest survival benefit depending on the stage, but more than half of patients relapse. Given this need for improvement, over the last years different targeted therapies have been evaluated in early-stage NSCLC with no survival benefit in unselected patients. However, the identification of reliable predictive biomarkers to these agents in the metastatic setting, the design of molecularly-oriented studies, and the availability of novel potent and less toxic agents opened the way for a novel era in early stage NSCLC treatment. In this review, we will discuss the current landscape of targeted therapeutic options in early NSCLC.

## 1. Introduction

Non-small-cell lung cancer (NSCLC) represents roughly 85% of lung cancers, with an incidence that keeps rising across the globe [1]. The treatment landscape of metastatic NSCLC has considerably improved over the last two decades due to a better understanding of cancer biology [2]. The introduction in clinical practice of several new and more effective molecules has led to a consistent improvement in overall survival (OS) and quality of life (QoL) in advanced/metastatic disease [3].

Oncogenic drivers have indeed reshaped the therapeutic algorithm for metastatic NSCLC, making us wonder whether they could also play a role in early disease. Roughly a quarter of patients are diagnosed with disease amenable to potentially radical surgery [3]

In spite of this, five-year survival rates for fully resected stage I disease range from 50 to 70% while lying between 10 and 30% for stage IIIA NSCLC [4].

**Figure 1:**
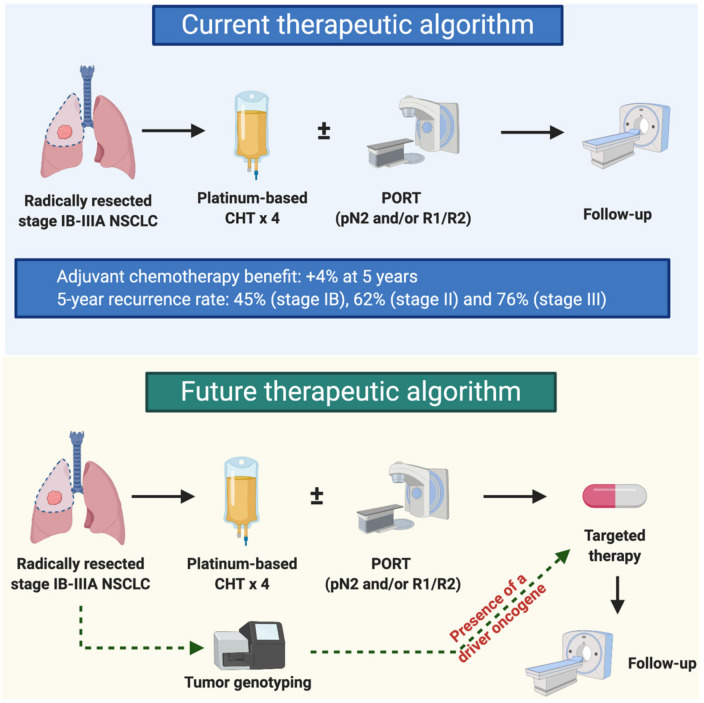
Potential role of targeted therapies in the adjuvant setting. Post-operative platinum-based chemotherapy (CHT) in stage IB-IIIA resected non-small-cell lung cancer (NSCLC) has been associated with a 4% survival benefit at 5 years [7]. According to the Lung Adjuvant Cisplatin Evaluation (LACE) study, 5-year recurrence rates range from 45% in stage IB to 76% in stage III after adjuvant chemotherapy [8]. Post-operative radiotherapy (PORT) is currently recommended in patients with pathologic N2 (pN2) disease and in those with microscopic (R1) or macroscopic (R2) residual disease.

Adjuvant chemotherapy has shown a modest survival benefit (with an absolute increase in survival of 4% at five years), but, depending on the stage [5,6], more than half of patients still relapse [7]. Similarly, a neoadjuvant approach yields a 5% absolute benefit on five-year survival [8], leaving room for improvement. The question of whether targeted therapy could fill this unmet medical need is far from new. Over the last several years, different targeted therapies have been evaluated in early stage NSCLC with no survival benefit in unselected patients [9,10,11,12]. However, the identification of reliable predictive biomarkers to these agents in the metastatic setting, the design of molecularly-oriented studies, and the availability of novel potent and less toxic agents paved the way for a novel era in early stage NSCLC treatment (Figure 1). In this review, we will discuss the current landscape of targeted therapeutic options being investigated in early NSCLC.

## 2. Epidermal Growth Factor Receptor (EGFR) mutations

In EGFR mutant NSCLC, the most common oncogenic driver targeted today, the question of adding a tyrosine kinase inhibitor (TKI) or replacing chemotherapy with one is not new. Before EGFR mutations were identified as the major determinants of efficacy to first-generation EGFR TKIs in 2004 [13,14], two randomized phase III trials assessed the impact of erlotinib (RADIANT) [9] and gefitinib (BR19) [10] in unselected stage IB-IIIA NSCLCs. The RADIANT trial enrolled patients with fully resected stage IB to IIIA NSCLC and confirmed tumor EGFR expression by immunohistochemistry (IHC) or fluorescent in situ hybridization (FISH). Patients were randomized in a 2:1 ratio to receive daily erlotinib for two years or placebo. When indicated, patients received adjuvant chemotherapy before starting the study therapy. The primary endpoint of improved DFS was not met. It is noteworthy that only 163 out of 973 patients recruited in the study harbored an exon 19 deletion or L858R EGFR mutation. In this specific subgroup of EGFR-driven diseases, DFS was superior with erlotinib (HR, 0.61; 95% CI, 0.384–0.981; *p* = 0.0391). This difference could not be retained as statistically significant, given the hierarchical testing that allowed assessment of secondary endpoints only if the primary endpoint was statistically significant. The follow-up was too short at the time of the analysis to properly assess survival differences. The phase 3 BR19 study assessed the role of gefitinib as adjuvant therapy for up to two years versus placebo in patients with completed resected stage IB to IIIA NSCLC. At a median follow-up of 4.7 years among 503 patients, there was neither DFS (1.22, 95% CI 0.90–1.71) nor OS (HR 1.24, 95% CI 0.94–1.64) benefit in the experimental arm. The trial was closed early. No benefit was also observed in the small subgroup of patients harboring EGFR mutations in terms of both DFS (HR, 1.84; 95% CI, 0.44 to 7.73; *p* = 0.395) and OS (HR, 3.16; 95% CI, 0.61 to 16.45; *p* = 0.15) [10].

Following the successful experience in advanced/metastatic setting with multiple EGFR TKIs approved in molecularly selected patients [15,16,17,18,19] different studies have sought to demonstrate a survival advantage with the use of these agents as adjuvant therapy in EGFR-mutated radically resected NSCLCs (Table 1). Nevertheless the role of co-mutations remains unclear [20].

The SELECT trial is a single-arm phase 2 trial and was the first to test the efficacy of adjuvant erlotinib in resected EGFR-mutated NSCLC. One hundred patients with stage IA to IIIA EGFR mutant NSCLC were given erlotinib for up to two years after completing standard adjuvant therapy. The 2-year DFS was 88%, and the authors concluded that this was an improvement compared to historical matched controls, which had a two-year DFS of 76%. Furthermore, the median time to recurrence was 25 months after discontinuing erlotinib [11]. Since then, a number of randomized adjuvant targeted therapy trials have been published.

The CTONG1104/ADJUVANT trial is a phase 3 study that compared the standard cisplatin-vinorelbine chemotherapy to gefitinib, a first-generation EGFR TKI in fully resected stage II to IIIA EGFR mutant NSCLC [12]. Chemotherapy was administered for four cycles, while gefitinib was given until progression for up to two years. The primary endpoint was disease-free survival (DFS), while OS was a secondary endpoint. At a median follow-up of 36.5 months, the experimental arm yielded a superior DFS compared to chemotherapy (28.7 versus 18.0 months), with a hazard ratio (HR) of 0.60 (95% CI 0.42–0.87, *p* = 0.0054). The mature OS results were presented at the American Society of Clinical Oncology (ASCO) 2020 Meeting, with a median follow-up of 76.9 months. The median OS was not statistically different, at 75.5 months in the gefitinib arm and 79.2 months in the chemotherapy arm (HR 0.92, 95% CI 0.62–1.36). Furthermore, only 51.5% of patients in the chemotherapy arm were exposed to a TKI at progression [13].

The EVAN trial is a phase 2 randomized trial on a smaller cohort of 102 patients with fully-resected stage IIIA EGFR-mutant NSCLC [21]. Patients were randomized between adjuvant chemotherapy for four cycles and erlotinib for up to two years. The primary endpoint was two-year DFS, while OS was a secondary endpoint. At a median follow-up of 33 months, the two-year DFS was 81.4% in the erlotinib group and 44.6% in the chemotherapy arm (RR 1.823, 95% CI 1.194–2.784, *p* = 0.0054). While these results are promising, the sample size was small, and OS data are not yet available.

Recently, the ADAURA trial was presented at the ASCO 2020 Meeting [22]. It evaluated the impact of adjuvant osimertinib, a third-generation EGFR TKI, compared to placebo in fully-resected stage IB to IIIA (TNM 7) EGFR-mutant NSCLC. Patients in both arms were eligible for chemotherapy, but the trial was not stratified based on whether it was administered. The experimental arm received osimertinib until progression or up to three years. The primary endpoint was once again DFS, among the stage II to IIIA patients, while DFS in the intention-to-treat population and OS were among the secondary endpoints. The results presented at the ASCO Meeting were from an off-protocol interim safety analysis after the data safety monitoring board asked to unblind the trial due to a very strong signal favoring the osimertinib arm. At this preliminary analysis, with a median follow-up of 22 months, the DFS for stage II to IIIA patients was not reached in the osimertinib arm and 20.4 months in the placebo arm, with an HR of 0.17 (95% CI 0.12–0.23, *p* < 0.0001). While the HR of DFS is certainly impressive, there is much controversy about whether these immature data should lead to a change in practice, as the real question is whether patients will live longer if treated earlier. Furthermore, at the ASCO 2020, the final analysis of the CTONG1104/ADJUVANT trial was presented, showing that treating EGFR positive patients with EGFR TKI delayed the relapse but did not translate into OS benefit [12]. Moreover, the ADAURA trial doesn’t address the question about the importance and need for adjuvant chemotherapy in these patients, as the majority of them received it before being randomized in the trial.

Probably the largest effort to address the role of EGFR TKI in the adjuvant setting is the ALCHEMIST trial (NCT02194738). Patients with stage IB to IIIA after radical surgery will get molecular analysis, and patients whose tumor harbors an EGFR mutation will enter the EGFR mutation substudy. It is aiming to recruit 410 patients and will randomly assign patients to erlotinib for two years versus placebo. The primary endpoint is OS.

Neoadjuvant therapy has the potential to facilitate surgery by shrinking the tumor. A phase II single-arm study assessing the impact of 28 days of neoadjuvant gefitinib in stage I NSCLC found a 50% response rate among patients whose tumors harbored EGFR mutations. There was no safety signal for increased risk of surgery. Upon histologic analysis, there was more fibrotic scar tissue, lower cell proliferation, and residual tumor cells were concentrated in fibrous stroma with lymphocytic infiltration [23,24]. Three small phase II trials evaluated neoadjuvant erlotinib among patients with stage IIIA EGFR-mutant NSCLC. In spite of a response rate of 58%, the first failed to show a survival benefit compared to non-EGFR-mutated patients receiving chemotherapy [25]. The second was a single-arm trial and reported a 42% response rate, with 21% downstaging to T0-3 N0 M0. On a pathological level, 50% of patients had a partial response, while 50% had stable disease [26]. The third study compared neoadjuvant erlotinib among 15 patients whose tumors had EGFR mutations to chemotherapy in 16 patients without these alterations [27]. The authors report a trend towards better response rate, pathological response rates, and overall survival. Given the small number and heterogeneous prognosis inherent in oncogenic driven NSCLC, no conclusions can be drawn. One potential pitfall is disease flare after TKI interruption, and this will need to be evaluated in larger prospective trials [28]. The ongoing phase II EMERGING trial is comparing neoadjuvant erlotinib to cisplatin-gemcitabine in patients with stage IIIA EGFR-mutant NSCLC (NCT01407822). A neoadjuvant phase III trial of gefitinib versus carboplatin and vinorelbine among patients with stage II-IIIA EGFR-mutant NSCLC is planned (NCT03203590).

After the impressive early results of the adjuvant ADAURA trial, the neoADAURA (NCT04351555) trial will follow. It is a phase III trial that will compare neoadjuvant osimertinib, with or without chemotherapy, to chemotherapy alone in resectable NSCLC patients. The primary endpoint will be major pathological response rates, while OS and DFS will be among secondary endpoints. A phase II trial is ongoing (NCT03433469).

## 3. Anaplastic Lymphoma Kinase (ALK) Gene Fusions

ALK rearrangements are detected in 2 to 7% of NSCLC patients. Interestingly, this comprises under 5% of resected NSCLCs but up to 19% of stage IV cancers. This could possibly be due to the biology of ALK driven tumors, with rapid proliferation and spread. It is interesting to note that this contrasts with EGFR mutations, which are not stage dependent [29,30,31].

Given the infrequency of ALK translocations in localized NSCLC, it is no surprise that there are fewer clinical trials assessing ALK inhibitors in this setting. Nonetheless, two adjuvant phase III trials are ongoing. The aforementioned ALCHEMIST trial (NCT02194738) has an arm randomizing patients with stage IB to IIIA (TNM7) fully-resected ALK-driven NSCLC to observation versus crizotinib for up to 24 months after completing standard therapy, including chemotherapy and radiotherapy, where indicated. The primary endpoint is OS, and DFS is among secondary endpoints. The second, more recent, phase III multicenter randomized adjuvant trial, ALINA (NCT03456076), is comparing alectinib to standard of care in stage IB-IIIA (TNM 7) fully resected ALK-rearranged NSCLC. This trial excludes patients with N2 stage IIIA cancer who could be candidates for postoperative radiotherapy in some centers, as this could represent a confounding factor in a high-risk group. Alectinib is administered for up to 24 months, while the control arm receives four cycles of platinum-based chemotherapy. The primary endpoint is DFS, while OS is among secondary endpoints.

Finally, a multicenter phase II trial will soon begin recruiting patients with stage IB to IIIB resectable NSCLC with a variety of oncogenic drivers, assessing the effect of eight weeks of neoadjuvant therapy followed by the possibility of adjuvant treatment with the same drug (NCT04302025). One arm will assess alectinib in this setting. The NCT03088930 trial is evaluating neoadjuvant crizotinib in a similar design.

## 4. Others Oncogenic Drivers

EGFR and ALK are two of the multitude of currently identified and targetable oncogenic drivers in NSCLC. As a reminder, in lung adenocarcinoma, targetable alterations comprise roughly half of all diseases, and the list of actionable therapeutic targets is constantly growing [32].

Front-line therapy for metastatic disease has largely shifted or is gradually moving towards a TKI approach among patients whose tumors harbor these alterations. To name a few, BRAF V600E mutations are successfully targeted by combined BRAF/MEK inhibitors, mainly dabrafenib and trametinib, with high response rates and relatively low toxicity [33]. Similarly, ROS1 rearrangements can be targeted with drugs, including crizotinib, ceritinib, lorlatinib, entrectinib, or repotrectinib [26]. MET alterations, including exon 14 mutations and amplifications, can be treated with a variety of TKIs, among the most common, crizotinib, capmatinib, and tepotinib [34,35,36,37]. NTRK fusions are a recent addition to this list, with impressive responses to entrectinib and larotrectinib [38,39]. RET fusion-positive NSCLC also appears to benefit from treatment with selpercatinib or praseltinib [40,41] The very common KRAS G12C mutation, hitherto considered undruggable, is now being targeted by small molecules, with the potential to dramatically alter the therapeutic landscape [42]. HER2 targeting appears to be moving forward in strides, with the antibody-drug conjugate, trastuzumab–deruxtecan, yielding impressive preliminary results [43]. It is important to be aware of the efficacy of these drugs, even if only in early phase trials for some, to understand the rationale for testing them in the adjuvant or neoadjuvant setting.

The abovementioned ALK trial (NCT04302025) also includes arms for ROS1 and NTRK, each to be treated by entrectinib following the same treatment pattern as the ALK arm, as well as BRAF V600E mutations to be treated by vemurafenib and cobimetinib. The primary endpoint is major pathological response, defined as 10% or lower of viable tumor cells, while OS and DFS are among secondary objectives.

A small phase II trial is ongoing, assessing the efficacy of six weeks of neoadjuvant crizotinib in patients with MET, ROS1, or ALK alterations and resectable stage IA-IIIA NSCLC (NCT03088930). Objective response rate is the primary endpoint while OS and DFS are among secondary endpoints.

These trials are summarized in Table 2. To our knowledge, no other phase II to III trials are ongoing in this setting.

## 5. Limitations of TKIs in the Early Stage Setting

To date, no data have proven that TKIs can be curative in NSCLC, and patients ‘compliance with TKI treatment could be lowered by the persistency of chronic adverse events due to long term use of TKIs in patients who are free from cancer [36,37]. In the metastatic setting, all TKIs eventually fail, through on- or off-target escape mechanisms. Similarly, treatment discontinuation can lead to a flare in tumor growth. The aim of TKI therapy in early-stage disease could comprise various options. Of course, the primordial question is whether treating microscopic residual disease could eliminate cells rather than simply suppress growth, thus increasing cure rates rather than just relapse-free survival. Only the overall survival of properly conducted trials will provide this answer. However, other goals could exist. In the neoadjuvant setting, for instance, the higher response rate of TKIs compared to chemotherapy could facilitate surgical management.

Under selective therapy-induced pressure, oncogenic-driven tumors can develop different resistant clones. While cell subpopulations develop a quiescent or dormant state of cell-cycle arrest when exposed to TKIs, some will acquire resistance alterations, whether through mutations or epigenetic changes [44].

The drug-tolerant cells are a reservoir for potential tumor growth and will lead to progression if they escape immune surveillance and proliferate. Unless we manage to reactivate quiescent cells selectively to target them, it is unlikely TKIs will be curative given this behavior [45].

Combinatory therapy targeting and inhibiting signal transduction and activator of transcription 3 (STAT3) and Src may potentially be more effective by reducing the level of lung cancer stem cells subpopulation

## 6. Future Directions and Challenges

As we await the results of ongoing and planned trials of TKIs in the localized NSCLC setting, the question of which patients to treat in the non-metastatic setting may emerge. Circulating tumor DNA (ctDNA) can detect minimal residual disease among patients with operated early-stage NSCLC [46]. Similarly, ctDNA has been used to identify acquired-resistance mechanisms to TKIs [41,42,47,48]. Such an approach could have potential implications for initiating therapy upon early disease detection rather than broadly among all operated patients. It could also be a tool to monitor patients on adjuvant therapy in order to detect early resistance or relapse. These questions and more will have to be revisited in light of results of neo(adjuvant) trials. Only time will provide answers about the best care for our patients.

At the ASCO 2020 Meeting, the ADAURA presentation may have provided a glimpse of the future for adjuvant treatment in oncogenic driven mutated NSCLC. The assumption that a TKI, proven to be very effective in the metastatic setting, could and will be even more effective in early stages has become prevalent among many oncologists. There may be significant implications if the follow-up data of the ADAURA trial and the readouts from the ALINA are very positive, albeit for surrogate endpoints for OS. Oncologists may be very tempted to emulate this early-stage TKI approach in less frequent mutations, such as NTRK or RET, arguing that large trials are not feasible given the rarity of these alterations. This will generate a debate on the appropriateness and validity of extrapolating results without a formal trial; however, it is undeniable that the oncology world has already, as a consequence of preliminary results, shifted toward wider molecular genomic sequencing in early-stage NSCLC.

## Figures and Tables

**Table 1:** Phase II/III studies with EGFR tyrosine kinase inhibitors (TKIs) in the adjuvant setting.

Study	Phase	Population	n	Arm(s)	Patients Receiving Adjuvant Chemotherapy (%)	Median DFS (mos)	2-Year DFS	3-Year DFS	Median OS (mos)
**RADIANT** [9]	III	IB-IIIA NSCLCs, EGFR-positive by IHC and/or FISH	623 vs. 250	Erlotinib for 2 years vs. placebo	50.6% vs. 57.1%	50.5 vs. 48.2 (HR 0.90)	75% vs. 54%	N.R.	Not reached vs. Not reached (HR 1.09)
**BR19** [10]	III	IB-IIIA NSCLCs	251 vs. 252	Gefitinib for 2 years vs. placebo	17% vs. 17%	4.2 years vs. Not reached (HR 1.22)	N.R.	N.R.	5.1 years vs. Not reached (HR 1.24)
**SELECT** [11]	II	IA-IIIA EGFR-mutated NSCLC	100	Erlotinib for 2 years	N.R.	Not reached	88%	N.R.	Not reached
**CTONG1104** **ADJUVANT** [12]	III	II-IIIA EGFR-mutated NSCLC	111 vs. 111	Gefitinib for 2 years vs. vinorelbine/cisplatin	0% vs. 100%	30.8 vs. 19.8 (HR 0.56)	N.R.	39.6% vs. 32.5%	75.5 vs. 62.8 (HR 0.92)
**EVAN** [20]	II	IIIA EGFR-mutated NSCLC	51 vs. 51	Erlotinib for 2 years vs. vinorelbine/cisplatin	0% vs. 100%	42.4 vs. 21.0 (HR 0.268)	81.4% vs. 44.6%	54.2% vs. 19.8%	Not reached vs. Not reached (HR 0.165)
**ADAURA** [21]	III	IB-IIIA EGFR-mutated NSCLC	339 vs. 343	Osimertinib for 3 years vs. placebo	55% vs. 56%	Not reached vs. 20.4 (HR 0.17) *	90% * vs. 44% *	80% * vs. 28% *	Not reached vs. Not reached (HR 0.40) *

* Intention-to-treat (ITT) population (stage II-IIIA NSCLC). Abbreviations: IHC, immunohistochemistry; FISH, fluorescent in situ hybridization; DFS, disease-free survival; OS, overall survival; mos, months; HR, hazard ratio; N.R., not reported.

**Table 2:** Ongoing phase II–III neoadjuvant and adjuvant trials with tyrosine kinase inhibitors (TKIs).

Trial	Phase	Design	Population	Arm(s)	Primary Outcome	Clinical Trial Identification
**ALCHEMIST**	III	adjuvant	IB-IIIA NSCLCs, EGFR-mutated NSCLC	erlotinib for 2 years vs. placebo	OS	NCT02194738
**ALCHEMIST**	III	adjuvant	IB-IIIA NSCLCs, ALK-rearranged NSCLC	crizotinib for 2 years vs. placebo	OS	NCT02194738
**ALINA**	III	adjuvant	IB-IIIA NSCLCs, ALK-rearranged NSCLC	alectinib for 2 years vs. chemotherapy	DFS	NCT03456076
**EMERGING**	II	Neoadjuvant + adjuvant	IIIA EGFR-mutated NSCLCs	erlotinib for 6 weeks then 1 year post-op vs. cisplatin-gemcitabine	ORR	NCT01407822
**NCT03203590**	III	neoadjuvant	II-IIIA EGFR-mutated NSCLC	gefinitib for 8 weeks vs. carboplatin-vinorelbine	2 year DFS	NCT03203590
**NeoADAURA**	III	neoadjuvant	II-IIIA EGFR-mutated NSCLC	osimertinib +/− platinum-pemetrexed vs. platinum-pemetrexed	MPR	NCT04351555
**NCT04302025**	II	Neoadjuvant +/− adjuvant	IB-IIIB NSCLC with altered ALK, ROS1, NTRK or BRAF	8 weeks neoadjuvant +/− adjuvant with alectinib, entrectinib or vemurafenib+cobimetinib	MPR	NCT04302025
**NCT03088930**	II	neoadjuvant	IA-IIIA NSCLC with altered MET, ROS1 or ALK	crizotinib for 6 weeks	ORR	NCT03088930

Abbreviations: DFS, disease-free survival; OS, overall survival; MPR, major pathological response; ORR, objective response rate.
